# Evaluation of Several Computer Vision Feature Detectors/Extractors on Ahuna Mons Region in Ceres and Its Implications for Technosignatures Search

**DOI:** 10.3390/vision6030054

**Published:** 2022-08-31

**Authors:** Gabriel G. De la Torre

**Affiliations:** Neuropsychology and Experimental Psychology Lab, Campus Rio San Pedro, University of Cadiz, 11510 Puerto Real, Spain; gabriel.delatorre@uca.es; Tel.: +34-646287398

**Keywords:** technosignature, Ceres, computer vision, perception, cognitive bias

## Abstract

Ahuna Mons is a 4 km particular geologic feature on the surface of Ceres, of possibly cryovolcanic origin. The special characteristics of Ahuna Mons are also interesting in regard of its surrounding area, especially for the big crater beside it. This crater possesses similarities with Ahuna Mons including diameter, age, morphology, etc. Under the cognitive psychology perspective and using current computer vision models, we analyzed these two features on Ceres for comparison and pattern-recognition similarities. Speeded up robust features (SURF), oriented features from accelerated segment test (FAST), rotated binary robust independent elementary features (BRIEF), Canny edge detector, and scale invariant feature transform (SIFT) algorithms were employed as feature-detection algorithms, avoiding human cognitive bias. The 3D analysis of images of both features’ (Ahuna Mons and Crater B) characteristics is discussed. Results showed positive results for these algorithms about the similarities of both features. Canny edge resulted as the most efficient algorithm. The 3D objects of Ahuna Mons and Crater B showed good-fitting results. Discussion is provided about the results of this computer-vision-techniques experiment for Ahuna Mons. Results showed the potential for the computer vision models in combination with 3D imaging to be free of bias and to detect potential geoengineered formations in the future. This study also brings forward the potential problem of both human and cognitive bias in artificial-intelligence-based models and the risks for the task of searching for technosignatures.

## 1. Introduction

Ceres is the closest dwarf planet to the Sun. The surface of Ceres contains water ice and various hydrated minerals. There is an open debate in the scientific community about the presence of cryovolcanism. NASA’s Dawn mission observed Ahuna Mons, a 4 km high landform on Ceres interpreted to be a cryovolcanic dome [1]. Cryovolcanism is a form of volcanism involving water or other volatiles instead of silicate magmas [2]. Cryovolcanic domes have been detected on other planets and moons such as Titan, Europa, and Pluto.

**Figure 1:**
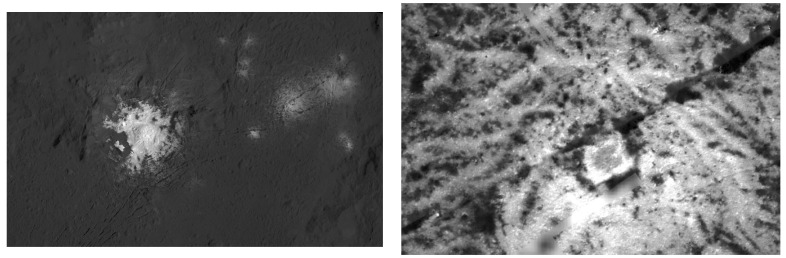
**Left**: Vinalia Faculae, Occator crater on Ceres. **Right**: Section image from original PIA21925. Original image credit: NASA/JPL-Caltech/UCLA/MPS/DLR/IDA/PSI.

Ahuna Mons is the youngest of these proposed cryovolcanic domes, dated at less than ~200 Ma old [3,4]. Even younger cryovolcanic features, as young as ~4 Ma, have been reported on the floor of Occator by [5]. The Occator crater (Ø 92 km, ~4 km deep) is one of the most intriguing surface features on Ceres because it contains what are called the bright spots on Ceres, which are very bright regions that clearly stand out from the relatively dark surroundings. These bright spots are known as faculae, and previous studies have reported that the faculae are mainly sodium carbonate structures [6] and are suggested to be significantly younger than the impact crater itself [5,7], although low altitude mapping orbit (LAMO) imaging by the Dawn probe was insufficient for a reliable age determination. Some apparently geometric formations in its interior have also been reported [8] (Figure 1).

**Figure 2:**
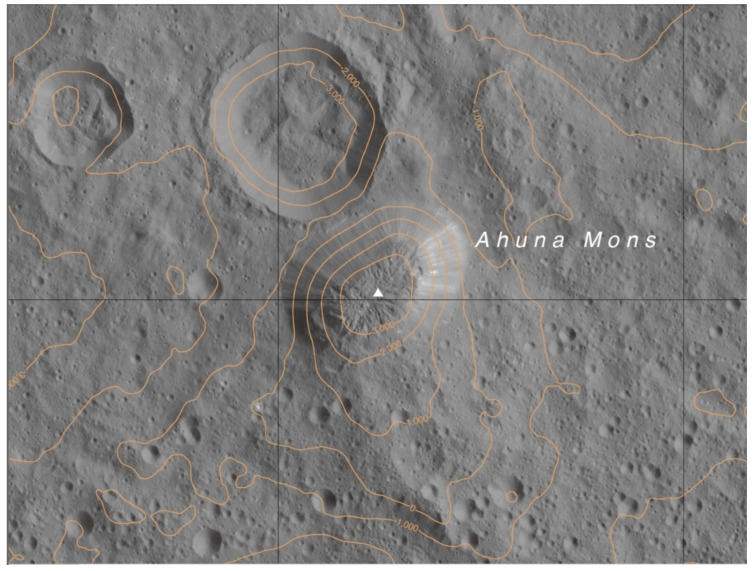
Altitude map of Ahuna Mons and surrounding area on Ceres, including Crater B next to it. Image credit: NASA/Ceres LAMO atlas. Ac-L-42-Ahuna Reprinted/adapted with permission from [9,10]. Scale 1:250,000.

According to [1] “the ~17-km-wide and 4-km-high Ahuna Mons has a distinct size, shape, and morphology (Figure 2). Its summit topography is concave downward, and its flanks are at the angle of repose. The morphology is characterised by (i) troughs, ridges, and hummocky areas at the summit, indicating multiple phases of activity, such as extensional fracturing, and (ii) downslope lineations on the flanks, indicating rockfalls and accumulation of slope debris”. However, this debris accumulation is not so clearly perceived in the space between Ahuna Mons and Crater B (NW side), ending in a very sharply defined contour, which extends beyond, to the south and north sides of it. It is believed that some form of material extruded at high viscosity is needed to explain the dome relaxation morphology [1].

However, opinions contrary to cryovolcanic hypotheses exist, stating that since those other cryovolcanic domes exist on moons around giant planets such as Jupiter, receiving heat from tidal friction is not possible on Ceres. Moreover, radiogenic material could not explain that heat, given the age of the solar system and the fact that no flow features or other morphological indicators for cryovolcanism have ever been found on the dome [11]. Another explanation for the features of Ahuna Mons is the presence of a salt dome, in a similar form to those found on Earth [12].

One particular feature that attracted our attention was the crater beside Ahuna Mons, identified by some authors [1] as Crater B. This crater possesses several characteristics and properties that sparked the idea among us regarding its relationship with Ahuna Mons, if any. Apparently, no relationship should exist between these two geological features, but if we look closely things change, at least perceptually.

## 2. Perception and Cognitive Bias

Visual perception allows us to interpret our environment. This process is based on the transduction process of transforming light from the visible spectrum to nervous impulses and subsequent perceptions. It is well-known that what we ‘see’ does not necessarily correspond to the objective reality. This is due to perception being a complex process, where top-down and bottom-up mechanisms take place; with experience, expectations, and culture participate as the main actors. Visual experience often serves as a basic example of conscious experience. Several scientists and philosophers have focused solely on the study of visual percepts, as a means of identifying the minimal set of neural events required to elicit a conscious mental experience [13].

Cultural values, practices, and beliefs have a critical role in psychological [14] and neurobiological processes [15,16], underlying a wide range of behaviour manifestations; this has been demonstrated in several studies [17,18,19]. This not only affects psychological or behavioural aspects, but also cognitive processes and performance, or, more accurately, job and scientific performance. For example, in a recent study within the field of geology, ref. [20] tested how subjective bias in a fracture data collection has implications on the validity or reliability of the models that the data inform, such as the derived fluid flow parameters, rock strength characteristics, or paleo-stress conditions, observing considerable variability between participant interpretations. This assumes that the cognitive style of the participant is more important than experience, in how a participant interprets the studied media, i.e., the fracture network [20].

Cognitive style refers to the fact that individuals have habitual ways of performing tasks associated with cognitive processes such as attention, problem solving, decision-making, and interacting with others [21,22]. On the one hand, cognitive styles can have an impact on how people respond to stimuli and make decisions. On the other hand, our perceptual and other cognitive functions are determined by our physiology and neural circuits, limiting our comprehension of reality and constructing one as we give effort, attention, and intention to specific stimuli in our environment. These phenomena are frequent sources of perceptual and attention errors, which usually pass inadvertently in front of our eyes. For example, in cognitive psychology, we know that when people perform a selective looking or searching task by devoting attention to some aspects shown on a screen, while ignoring others, they often fail to notice unexpected information that may happen in that same display. This trend is called ‘satisfaction of search’, meaning that people are less likely to search for additional targets once they have found their original target.

According to dual-process theory, decision-making involves two different types of cognitive processes: one is based on intuition (Type 1) and the other on deliberation (Type 2) [23,24,25]. People tend to fit their cognitive style to the task they perform; this also happens in workplace environments, thereby creating a modus operandi organisational culture [26], as has been tested before in different jobs [27].

All these parameters promote the appearance of cognitive bias, which is not exclusive of any given task but most certainly affects every human task, scientific or not. The implications for this are serious, as, in a cumulative way, this bias can direct our knowledge, strategies, and development toward certain paths, excluding others that are probably more accurate. We consider the particular case of Ahuna Mons a good example. Most of the initial effort and hypotheses have been focused on the cryovolcanism origin hypotheses, although some reasonable doubts linger against this hypothesis, as stated above [11]. We also consider that special attention has been given to the main object (Ahuna Mons), while not paying deeper attention to the surrounding objects (Crater B in this particular case). There are several similarities between these two structures that strike our attention as well. Crater B is very similar in shape, size, height/depth, diameter, but not in age. Crater B and Ahuna Mons also share some intriguing similar features that stick out when both elements (Ahuna Mons and Crater B) are superimposed. The first explanation after realising this, and based on our previous argument, is cognitive bias. Incomplete, imperfect perceptual and cognitive processing and the styles of the human brain may be the first options to understand these objective similarities.

## 3. Artificial Intelligence and Computer Vision Models

Fortunately, we now have useful technology in the form of artificial intelligence (AI), and, more precisely, computer vision technology, to compensate for this cognitive bias and try to elucidate these pattern coincidences, serendipities, and findings. As mentioned before, this could be helpful in many disciplines, both in the life sciences and physical sciences. These AI technologies, including supervised and non-supervised machine learning systems and computer vision models, are of special interest in the field of the search for technosignatures, where cognitive bias can be a problem. However, AI is not free of bias, which is a topic of current research, since it may happen that AI models could suffer from the same perceptual and cognitive biases that humans present. Another possibility is that these AI models could bring us to the point of confronting us with a result that we are not ready to accept or understand. This is the case of a recent experiment, where humans and AI models were compared when looking for geometric patterns on Ceres (Vinalia Faculae in the Occator crater). The results of this research showed that both humans and AI-supervised machine learning models identified geometric patterns in one particular feature in this region (a square inside a triangle (Figure 1)) [8]. Supervised deep learning models where the experimenter has to feed previous sets of stimuli are sensible to bias, while simpler computer vision/feature detection models represent a very efficient, fast, and free-of-bias strategy. Finally, unsupervised deep learning models are also mostly free of bias, but since they rely on untagged non-specified data to find patterns, the computing cost and time results much higher.

**Figure 3:**
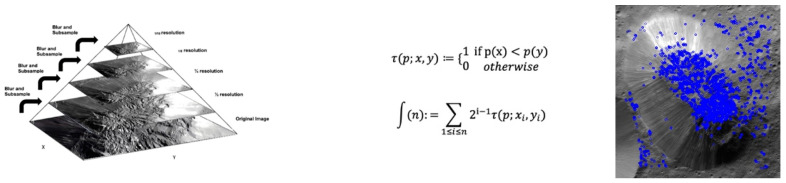
**Left:** Multiscale signal representation or image pyramid for Ahuna Mons. **Centre:** BRIEF binary feature descriptor. p(x) or p(y) are the intensity of image pixel (p), respectively, at a point x and y. In the case where the pixel at point y is greater than the pixel at point x, it is marked as 1 at points x and y; otherwise it is marked as 0. This is done for n location pairs of image p, *n* being the length of the binary feature vector. **Right**: ORB keypoint analysis for the Ahuna Mons image.

**Figure 4:**
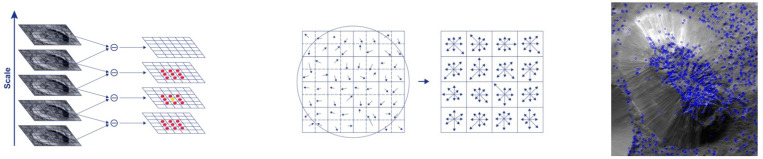
**Left:** After the Gaussian blur operator is applied for every pixel, we obtain the difference of Gaussians (DoG) (right), which will be helpful to identify the keypoints (red dots) of images of both Crater B and Ahuna Mons. **Centre:** Each keypoint has a location, scale, and orientation and is computed as a descriptor for the local image region about each keypoint that is highly distinctive and as invariant as possible (angle and luminosity). The keypoint descriptor is obtained using set of 16 histograms for the 4 × 4 grid of image with eight orientation bins for each grid item in the direction of candidate orientation, resulting in a feature vector of 128 units in size. **Right:** Keypoint dot analysis for the SIFT algorithm on the sample Ahuna Mons image. Left and centre images reprinted/adapted with permission from [28,29].

**Figure 5:**
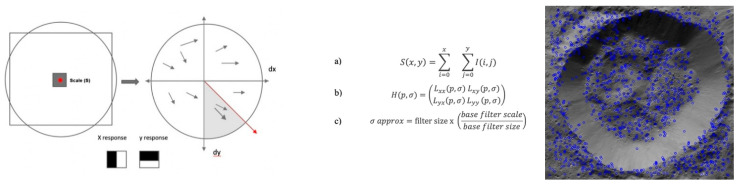
**Left:** The SURF algorithm has three main parts: point of interest detection, description, and matching. SURF uses square-shaped filters as an approximation of Gaussian smoothing and detects scale invariant points of interest (red dot), and DoG is calculated by rescaling the image progressively. SURF first calculates the Haar-wavelet responses in the x and y directions, in a circular neighbourhood of radius 6 s around the keypoint, with s representing the scale at which the keypoint was detected. **Centre:** First (top equation) the integral image is used for calculating the sum of values (pixel values) in a given image and represents the sum of all pixels in the input image I within a rectangular region formed by the origin and x. Next, the image is filtered by a Gaussian kernel (middle equation), so, for given a point X = (x, y), the Hessian matrix H (x, σ) is in x at scale σ. Lxx (x, σ) is the convolution of the Gaussian second-order derivative with the image I at point x and, similarly, for Lxy (x, σ) and Lyy (x, σ). Images are, therefore, repeatedly smoothed with a Gaussian filter and subsampled to the next higher level of the Gaussian pyramid (down equation). **Right:** Keypoint dot analysis for Crater B.

Computer vision is a form of artificial intelligence that trains computers to interpret reality; it has been an active area of research for decades. Common goals include the detection, recognition, and identification of objects or scenes within images or videos. There are several types of feature descriptors used in computer vision. Some of the most popular types of features include corner, blob, and feature descriptors including the techniques scale invariant feature transform (SIFT), speeded up robust features (SURF), oriented FAST and rotated BRIEF (ORB), and the Canny edge detector (Canny) (Figure 3, Figure 4 and Figure 5).

Over the last decade, the most successful algorithms to address various computer vision problems have been based on local, affine-invariant descriptions of images [30].

## 4. Methods

Several computer vision/feature detectors including SURF, SIFT, ORB, and Canny edge were used to compare Ahuna Mons and its nearby large crater (Crater B). As a control test, we also compared Ahuna Mons with another similar crater to Crater B. This control crater was a large crater around the Equator of Ceres; the experimental and focus of this research was Crater B, the large crater besides Ahuna Mons. We intentionally avoided using deep learning techniques because those models depend on previous training with pre-existing datasets, and this might represent a potential source of bias. Traditional computer vision algorithms such as the ones we used in this experiment represented a more efficient alternative. Algorithms such as SIFT and even simple colour thresholding and pixel counting algorithms are not class-specific, that is, they are very general and perform the same for any image with fewer coding lines than deep learning models [31]. Finally, for the image matching task, local descriptors from both images were matched through comparison performed by computing the Euclidian distance between all potential matching pairs by k-nearest neighbours’ algorithm (KNN). Nearest neighbour distance ratio matching criterion was used to minimize mismatches, combining this with RANSAC-based technique [32]. Efficiency in the task is usually measured by match ratio and time-related efficiency.

**Figure 6:**
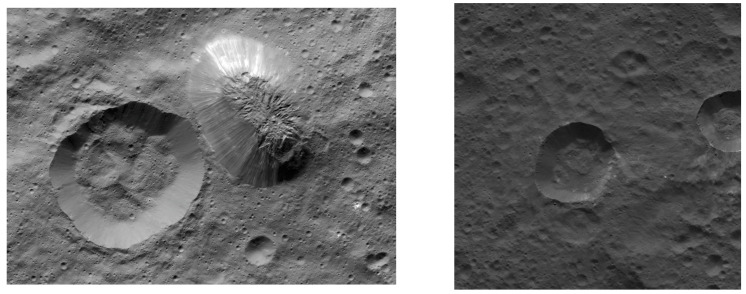
**Left:** Ahuna Mons and Crater B as seen in this mosaic of images from NASA’s Dawn spacecraft from 385 km (240 miles) above the surface, in December 2015. The resolution of the image is 35 m (120 feet) per pixel. (PIA20348). **Right**: Equator crater in Ceres used as control image for baseline computer vision comparison analysis with Ahuna Mons. (PIA20677). Images credit: NASA/JPL-Caltech/UCLA/MPS/DLR/IDA (130–140 m/px).

Preliminary observational analysis of Ahuna Mons and Crater B data resulted in similar characteristics including the contour and shape (oval), average depth (≅4 km), diameter (≅17 km), and several surface features that are more relevant when the two geological objects are superimposed at a specific angle (left 90° for Ahuna Mons over Crater B). According to [1], the 17 km crater B has an estimate age of 160 ± 30 million years using the Lunar-Derived Model or 70 ± 20 million years using the Asteroid-Derived Model. Ahuna Mons has an upper limit of 240 million years of age, but it is difficult to estimate because it has so few impact craters on it, so it could actually be much younger [33]. Despite the popular cryovolcanism origin hypothesis for Ahuna Mons, there is no appreciable debris in the strait between both formations on the NW side. In this project, we used Open Cv2 (*Open-Source Computer Vision Library)* to extract features from both the Ahuna Mons–Crater B and Ahuna Mons–Equator Crater pair of images (Figure 6) through SIFT, SURF, ORB, and Canny edge feature-detection/extraction techniques and matched them across to stitch the images together. We also performed 3D object analysis derived from imaging data of both structures and tested how both objects fit in tridimensional space using specific 3D rendering software 3.0. (Blender 3D, 2021).

## 5. Results

As SIFT features were obtained through detecting extrema points using the scale-space to make it scale invariant; we set a value of 1.6 Gaussian sigma to obtain different zoomed-level images at each octave and then applied keypoint localization to eliminate poor keypoints through a contrast threshold of 0.04, so that only the higher related pixels would be retained and less similar pixels would be ignored. We kept the edge threshold parameter at 10, so that larger area of the image could be processed. A local orientation histogram from gradient orientations of the sample points was obtained, and the highest peak in the histogram was taken as a candidate orientation, to make the feature descriptor a rotation invariant. Finally, a keypoint descriptor was obtained by using a set of 16 histograms for the 4 × 4 grid of the image, with eight orientation bins for each grid item in the direction of candidate orientation, thus giving a feature vector size of 128. In the progression of the pyramid for the scale invariance processing of the images, we kept three layers in the octave, per the findings of [28], and the features were ranked, per the policy of equal weightage (0 n features), to obtain a larger number of feature points. A summary of the hyper parameter explanations is included in Table 1.

SURF, as mentioned before, uses the determinant of Hessian matrix-based keypoints; the feterminant of Hessian is calculated by applying convolution with a Gaussian kernel and a second-order derivative. It is done efficiently by applying a LoG that approximates the convolution with box filters on the scale space pixels of the three octave layers of a pyramid containing four octaves. The pixels having a Hessian threshold greater than 100 were considered as winning pixels for further processing and obtaining descriptors. Since SURF is relatively slow in processing and obtaining feature descriptors, we used extended and upright as disabled, so that the speed could be increased by decreasing the feature size to 64 and ignoring the orientation processing.

The ORB technique compares the pixel brightness level to its surrounding neighbours with 16 pixels in its circular vicinity, by classifying them into three categories, i.e., lower brightness than the pixel, higher brightness than the pixel, and a similar level of brightness as that of the sample pixel. In this way, we obtained keypoints where half of the comparing pixels (eight pixels) had either a greater or lower level of brightness and limited them to a maximum of 500 features to be retained. The sample pixel and the results were also scale invariant, as this technique also uses the pyramid images of different scaling increasing by the factor of 1.2 f on eight levels. For feature description, ORB uses the BRIEF algorithm that computes feature vectors with a 128–512-bit binary string. It selects a random pair of pixels with two dimensions, as given in the WTA K parameter that is drawn from the Gaussian distribution centering on the keypoint, and compares its brightness with the second random pair of pixels drawn from a Gaussian distribution centered on the first pixel. The feature value of 1 is assigned if the first pixel is brighter than the second, and otherwise it is 0; in this way, a vector of binary string is obtained as a feature vector for the descriptor with scoring based on the Harris score mechanism. Here, we kept the edge threshold and patch size of 31 as default and a FAST threshold of 20 to obtain descriptors.

The Canny edge algorithm detects numerous edges in an image using a multi-stage edge detector. The intensity of the gradients is obtained by using a filter based on the derivative of a Gaussian, as a Gaussian reduces the effect of noise in the image by smoothing over six octaves in the pyramid and four layers per octave. The only retained edge pixels have a greater value than the hysteresis thresholding of 0.01 for the corner and DoG on the gradient magnitude. On those edge images, we obtain Harris–Laplace feature detector points that apply the second-order derivative to get keypoints, and we limit them to be within a 5000-point range, to make sure it does not exceed the processing limitations. We obtained feature descriptors for those keypoints by using the BRIEF algorithm, which gives a descriptor of 32 bytes without using the orientation on each keypoint.

**Figure 7:**
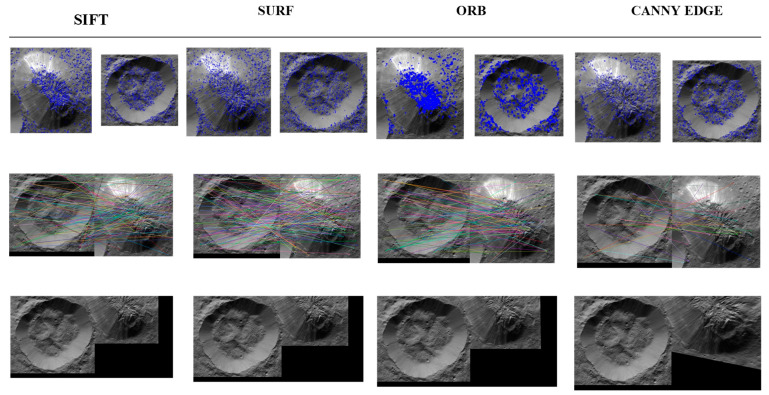
Comparative keypoint analysis (**top**), feature matching (**centre**), and image stitching (**bottom**) for best matched features for Ahuna Mons and Crater B using SIFT, SURF, ORB and Canny edge feature detection/computer vision models. Original images credit: NASA/JPL-Caltech/UCLA/MPS/DLR/IDA (130–140 m/px).

**Figure 8:**
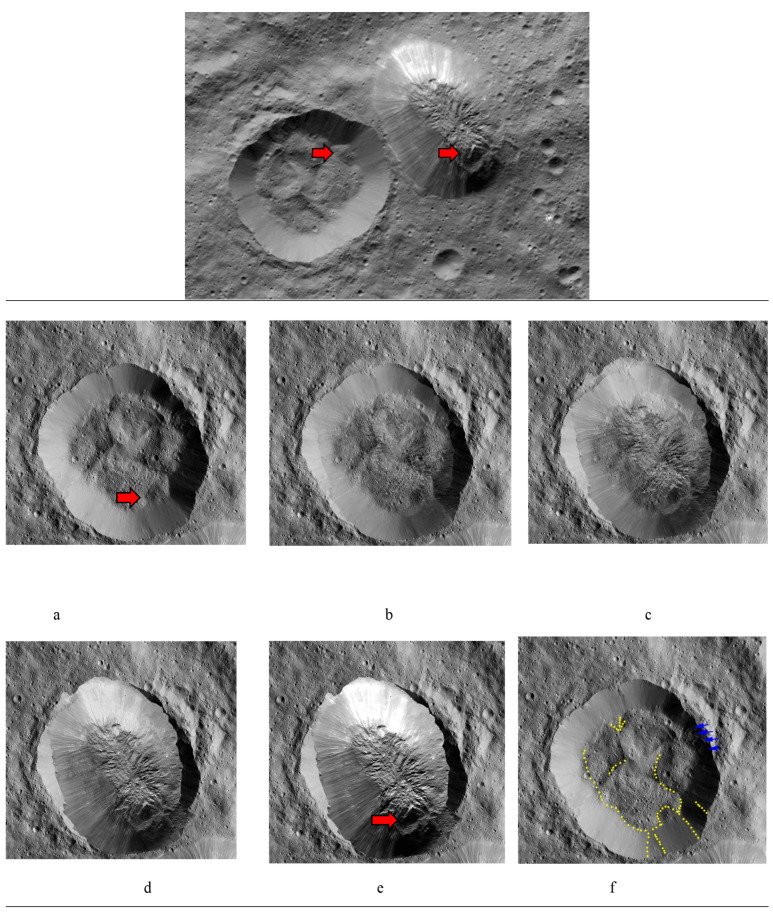
Qualitative 2D analysis of gradual overlapping of Ahuna Mons and Crater B. Top image: original image for reference. In red arrows, squared formations present in both Crater B and Ahuna Mons. Overlapping sequence of placing Ahuna Mons over Crater B making squared formations coincide. Crater B 100% (**a**), at 25% superimposed Ahuna Mons (**b**), at 50% (**c**), at 75% (**d**), and at 100% (**e**). (**f**) Image of Crater B shows some examples of the overlapping features between Ahuna Mons and Crater B (yellow dots) with Ahuna Mons (comparison of (**e**) and (**a**) images), especially the south region with particular squared formation. Blue arrows show how this side is also in both cases the more cratered one. Original images credit: NASA/JPL-Caltech/UCLA/MPS/DLR/IDA (130–140 m/px).

All matches were filtered by using the k-nearest neighbours algorithm (KNN) that compares the match distance differences among two images. The KNN match was then filtered through the Lowe test, for which the ratio was kept at 0.75 with a K value of 2 because we have two images to be compared. Among these techniques, the highest match ratio was for SURF and SIFT, because both of these techniques produce a large number of keypoint descriptors that cover almost every aspect of the image, as can be observed from the dot analysis of the images, i.e., the points are well-cluttered over the image in a large number. Based on runtime comparisons of the proposed techniques, Canny edge executes faster, as its implementation only requires it to extract edges through a mask kernel filter for convolution, whereas the other algorithms perform computations to detect numerous keypoints and their respective descriptors. The hyper parameters were tuned according to Ahuna Mons with Equator Crater images and later according to Ahuna Mons with Crater B images. The number of best features to retain for SIFT was 1500. A higher number would provide a greater number of features in the image with a contrast threshold of 0.04 and edge threshold of 10. Similarly, we have set the Hessian threshold to 200 with three octave layers. In the case of ORB, the number of features was set at a maximum of 5000, because it already produces a smaller number of features, so it is more accurate to set a large value for this technique. Moreover, its edge threshold is 31 with a patch size of 21. For the Canny Edge detector mask, the value of 50 was kept for the lower threshold of the gradient and 200 for the highest threshold along with the kernel filter, which was set to be a 3 × 3 mask as the default. All four techniques resulted in effectively finding matching features for Ahuna Mons and Crater B but not for Ahuna Mons and Equator Crater (Table 2 and Table 3). Among them, the Canny edge feature stood out as the best in terms of performance as well as the fastest execution runtime and match ratio in the experimental condition. Fastest performance and best-match ratio indicate less computing cost and the best results. The other techniques, although they offered good results, were noisier with irregular stitching results (Figure 7). The 2D qualitative analysis of overlapping both structures (Ahuna Mons and Crater B) also showed some possible coincidences, including a squared formation in the lower rim of Ahuna Mons and the lower slope of the crater (Figure 8).

## 6. 3D Analysis

**Figure 9:**
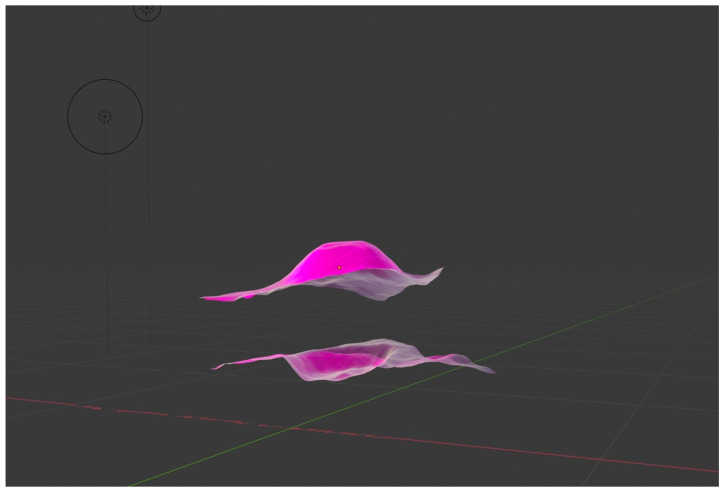
The 3D objects of Ahuna Mons (up) and Crater B (down) rendered in Blender 3D software.

**Figure 10:**
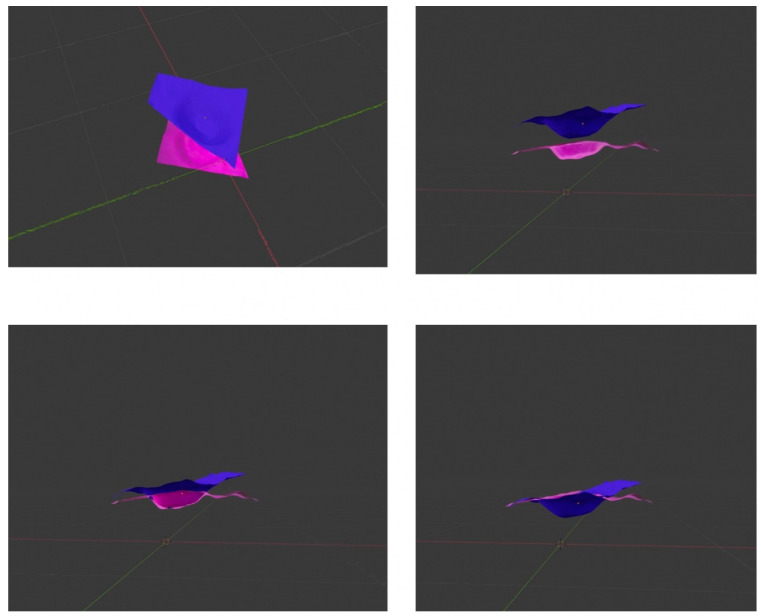
Mosaic of 4 3D blending images of inverted Ahuna Mons (blue) and Crater B (pink) 3D objects to show how Ahuna Mons fits inside Crater B.

In order to qualitatively compare the 3D objects of both structures, Ahuna Mons and Crater B, we developed 3D model objects of both using digital elevation models (DEM) of Ceres obtained from the Dawn mission (coordinate reference system (CRS): Equirectangular Ceres, Environmental Systems Research Institute (ESRI): 104972), later cutting out the areas of interest (Ahuna Mons and Crater B) using Geospatial Data Abstraction Library (GDAL) > clip raster by extent). Then, we imported the orthophotos or geometrically corrected images of both structures, obtained as well from Dawn mission data, into the open-source Geographic Information System (QGIS) and later into Blender open-source 3D suite, where they both were rendered (Figure 9). Interestingly, we could see how an inverted Ahuna Mons 3D model fits very well into Crater B (Figure 10). These results add to the previously findings obtained by feature detection, computer vision analysis, and qualitative overlapping 2D analysis, abounding again into a possible close relationship between both structures.

## 7. Discussion

According to our results, Ahuna Mons and Crater B are potentially very similar in several characteristics, and computer vision models confirm this similarity. The fact that these two formations share many objective data (similar height, diameter, shape, age, etc.) invites us to rethink the relationship between these two features and its geologic origin beyond the popular cryovolcanism hypotheses. We consider that the role and relationship of Crater B in the nature and origin of Ahuna Mons could be greater than expected, either geologically or by other unknown reasons. The results show that they also share patterns and features. One interesting possibility and potential to consider in future research is the ability of these computer vision models in combination with 2D and 3D modelling techniques and image processing to find patterns of geoengineered artificial formations.

We have seen that cognitive styles may produce cognitive bias in any workplace, and this situation may act as a filter, producing blindness to non-attended peripheral data, as has been shown before in cognitive psychology [34,35,36,37]. These results raise the question of cognitive bias in humans and AI models as a difficult outcome situation, because, in the future, these AI systems may reach a level of processing information and subsequent outcome for what we are not ready to understand. This may happen because these AI synthetic systems do not have our biological, cultural, and psychological biases and limitations. AI could take us to a place where none of our models fit in. Similarly, as found in Occator (Vinalia Faculae), spectroscopic investigation by spectral unmixing models confirms that Ahuna Mons is definitely a peculiar structure with respect to its composition; its flanks suggest a younger age and brighter material, possibly richer in carbonates compared to the surrounding areas [38]. Interesting features were detected in the Occator crater, Vinalia Faculae, which sparked the original frenzy over bright spots. Despite different studies defending the cryovolcano hypotheses, which necessarily assume this cryovolcanism to be a recent phenomenon [33] to fit the observed unique characteristics of Ahuna Mons, this structure remains as a very odd Ceres feature. This special characteristic makes Ahuna Mons, together with the Occator crater, two of the most interesting places for future exploration missions, posing them as excellent candidates for future landing sites, as has been stated before [39]. Our study adds a two-fold path for future research: one is a possible closer than previously expected relationship between Ahuna Mons and Crater B. The second is to open the debate for cases such as Ahuna Mons, where existing models hardly fit, and human expectations and workplace cognitive style may add some form of cognitive bias to the analysis task. AI technology could help overcome this bias but may also present some cognitive bias from those humans who developed them. The use of these computer vision and machine learning models are increasing in many different scientific disciplines, and they can either help us to understand and detect data collected in special complex cases such as this one, or take us to uncharted territory where our models and cognitive processing find a difficult-to-solve gap.

This is especially interesting in the area of the search for technosignatures. Technosignature means any measurable aspect that provides scientific evidence of past or present non-terrestrial technology. When scientists look for technosignatures in space, they basically have two options; i.e., one is a hypothesis-based search, according to models or expectations (e.g., search for intelligent radio signals), while the other is based on an opportunistic or serendipitous search (e.g., Oumuamua), also called non-canonical astrophysical phenomena [40]. The use of machine learning, with supervised or unsupervised models, is also increasing in this area of research. Research progress in this field needs to develop more sensitive and improved methods to detect possible technosignatures on the Moon [41,42], in the solar system [43] and beyond, and the progressive establishment of ever-stronger upper limits on specific signatures [44,45]. These upper limits will definitely involve new cognitive strategies, thereby expanding our understanding and promoting new paradigms. Convolutional neural network (CNN) models open new research possibilities beyond feature detection techniques, although they can also potentially be affected by bias.

According to our results and independently of the possible cryovolcanic or cognitive bias (either human- or AI-based) hypotheses, in the future, we have to be prepared for the possibility of a new form of technosignature, a natural-like object/phenomena where natural patterns are artificially or intelligently designed to be mostly indistinguishable from nature. We may need AI’s eyes and multidisciplinary teams to perceive, identify and understand them, whether they are star-like megastructures (Dyson spheres) [46] or mountains.

## Figures and Tables

**Table 1:** Cv2 hyper parameters used to instantiate cv2 algorithms for the analysis of the images.

Parameters	Explanation
Hessian threshold	Threshold value for filtering out the sharp keypoint detectors after applying Hessian on the output image.
n Octaves	Height of the octaves used to create pyramid for scale invariance.
n Octave layers	Number of layers used in each octave of the pyramid.
extended	Impacts the size of descriptor. False gives a 64D descriptor and true gives a 128D descriptor.
upright	Flag for computing orientation of the features to be included in the descriptor.
n features	Number of features that are to be included in the ORB descriptor.
Scale factor	Determines the factor by which the next pyramid level will decrease for scale invariance processing.
n levels	Gives the number of levels that the pyramid may have.
Edge threshold	Sets the number of pixels that are not to be considered in the descriptor.
First level	Level number that will contain the actual source image in the pyramid.
WTA_K	Impacts the dimension of the element in orient based BRIEF descriptor.
Score type	Takes the algorithm to be used for ranking and obtaining the best features for the target input. Default is kept to be HARRIS_SCORE.
Patch Size	Window size to be used for filtering a particular space (patch) in the image.
Fast threshold	Threshold used by the FAST algorithm to obtain the best feature keypoints.
contrast threshold	Threshold used by the SIFT feature to remove low contrast.
corn_thresh	Corner threshold value to filter whether the point is a corner.
DOG_thresh	Difference of Gaussians filter threshold for the selection of best points.
maxCorners	Limit on the maximum number of corners that an image may contain.
num_layers	Used by SIFT to determine the number of middle layers in an octave.
bytes	Sets the descriptor size for the BRIEF algorithm.
use_orientation	Flag to use orientation patterns/measure in the keypoint descriptor.

**Table 2:** Quantitative comparison and computational costs and time of the different feature detector descriptors for baseline analysis (Ahuna Mons vs. Equator Crater).

Method	Total Matches	Best Filtered Matches	Match Ratio	Execution Runtime (Seconds)
SURF	1781	3	0.1684	1.462
SIFT	1500	0	0.0000	0.699
ORB	200	0	0.0000	0.059
Canny edge	1018	2	0.1964	0.842

**Table 3:** Quantitative comparison and computational costs of the different feature detector descriptors (Ahuna Mons vs. Crater B).

Method	Total Matches	Best Filtered Matches	Match Ratio	Execution Runtime (Seconds)
SURF	1526	87	5.701	0.873
SIFT	1500	56	3.733	0.613
ORB	4682	52	1.111	0.739
Canny edge	1223	17	1.390	0.473

## Data Availability

Data from this study will be made available on reasonable request.
